# Application of Organic Acids and Essential Oils as Alternatives to Antibiotic Growth Promoters in Broiler Chickens

**DOI:** 10.3390/ani12172178

**Published:** 2022-08-25

**Authors:** Janghan Choi, Amit Kumar Singh, Xi Chen, Jirong Lv, Woo Kyun Kim

**Affiliations:** 1 Department of Poultry Science, University of Georgia, Athens, GA 30602, USA; 2 Nutribins, Walnut, CA 91789, USA; 3 DadHank (Chengdu) Biotech Corp, Chengdu 611130, China

**Keywords:** essential oils, organic acids, broilers, chickens, antibiotics alternatives, gut health, growth performance

## Abstract

**Simple Summary:**

It is essential to find alternatives to antibiotic growth promoters (AGP) after the restriction of AGP in the broiler industry. Organic acids (OAs) and essential oils (EOs) are considered promising alternatives to AGP to improve the growth performance and gut health of chickens due to their strong antimicrobial and antioxidant effects. In this study, OAs, EOs and their combinations were evaluated as AGP alternatives in broiler chickens. The supplementation of EOs improved feed efficiency in the starter phase compared to the control group, and the supplementation of OAs enhanced feed efficiency in the finisher phase compared to the control group with AGP. Hence, the supplementation of EOs and OAs could be potential AGP alternatives in the starter and finisher phase of broiler production, respectively.

**Abstract:**

This study was conducted to evaluate the effects of OAs and EOs on growth performance, serum biochemistry, antioxidant enzyme activities, intestinal morphology, and digestive enzyme activities to replace AGP in broilers. Six hundred one-day-old broilers were allotted to five treatments with six replicates: (1) negative control (NC; basal diet); (2) positive control (PC; NC + 50 mg/kg bacitracin methylene disalicylate); (3) organic acids (OA; NC + 2000 mg/kg OA); (4) essential oils (EO; NC + 300 mg/kg EO); and (5) OA + EO (NC + 2000 mg/kg OA + 300 mg/kg EO). In the starter phase, the PC, EO, and OA + EO groups had a significantly lower feed conversion ratio (FCR) compared to the NC group. While the final body weight (BW) of broilers fed OAs was similar compared to broilers fed PC (*p* > 0.1), the FCR of the OA group tended to be lower than the PC group on D 42 (*p* = 0.074). The OA group had the higher serum GLOB:ALB (albumin) and ileal villus height and crypt depth (VH:CD) ratios compared to the EO group. Thus, the supplementation of EOs and OAs could substitute AGP in the starter and finisher phase, respectively.

## 1. Introduction

Antibiotic growth promoters (AGP) are still widely used to improve gut health and growth performance in the global poultry industry, while many countries are trying to restrict the use of AGP in poultry feed [1]. The continuous and excessive use of AGP has been thought to decrease the efficacy of AGP and threaten public health by spreading antibiotic-resistant bacteria and genes in the poultry industry [2]. Removing AGP without appropriate replacement strategies may reduce production efficiency and cause food safety issues [3]. Hence, is essential to find effective strategies to replace AGP in the poultry industry [4,5].

The supplementation of individual or a combination of organic acids (OAs) and essential oils (EOs) has been studied and applied to improve the gut health and growth performance of animals [6,7]. OAs are known to improve the growth performance and gut health of broilers, mainly by modulating gut pH and exhibiting antimicrobial effects [8], and EOs are also known to have antimicrobial, antioxidative, and anti-inflammatory effects [9,10]. Furthermore, the use of a blend of diverse types of OAs and EOs could potentially show synergistic effects, enhancing gut health and exhibiting antimicrobial effects [11]. The effects of OAs and EOs on broilers vary depending on their dosages, sorts, and combinations. The effects of OAs, including benzoic acid, formic acid, and lactic acid, and EOs, including cinnamaldehyde, carvacrol, thymol, and eugenol and their blend, have not yet been investigated as AGP alternatives in broilers. Therefore, this study aimed to investigate the effects of OAs and EOs on growth performance, serum biochemistry, antioxidant enzyme activities, intestinal morphology, and digestive enzyme activities when used as antibiotic alternatives in broiler chickens.

## 2. Materials and Methods

### 2.1. Animals, Treatments, Diets, and Growth Performance

This present study was approved by the Institutional Animal Care and Use Committee at the University of Georgia. A total of 600 one-day-old broiler chickens were randomly distributed to 5 treatments with 6 replicates (20 broilers per pen (L: 1.52 m; W: 1.22 m; H: 0.61 m)) in a completely randomized design. The five treatments included (1) negative control (NC; basal diet); (2) positive control (PC; NC + 50 mg/kg bacitracin methylene disalicylate (BMD-50; Zoetis Products, Chicago Heights, IL, USA)); (3) organic acids (OAs; NC + 2000 mg/kg OA (Nutribins LLC, Walnut, CA, USA)); (4) essential oils (EOs; NC + 300 mg/kg EO (Nutribins LLC, Walnut, CA, USA)); and (5) OA + EO (NC + 2000 mg/kg OA + 300 mg/kg EO). The OAs contained benzoic acid, formic acid, and lactic acid, and the EOs included cinnamaldehyde, carvacrol, thymol, and eugenol. Corn-SBM basal diets for starter (D 0 to 14), grower (D 14 to 28), and finisher (D 28 to 42) were formulated to meet or exceed energy and nutrient requirements according to Cobb Broiler Management Guide (Cobb 2018) (Table 1). Feed additives (BMD-50, OAs and EOs) were included in the filler part with sand to obtain the desired concentrations of each additive in the feed. During the experimental period, chickens had free access water and feed, and temperature and light were controlled according to Cobb Broiler Management Guide (Cobb 2018). Broilers and their living conditions were monitored twice a day. Pen body weight and feed disappearance were recorded on D 14, D 28, and D 42 to calculate average daily gain (ADG), average daily feed intake (ADFI), and feed conversion ratio (FCR) for each feeding phase.

### 2.2. Sample Collection

On D 35, blood samples (5 mL) were collected from one randomly selected bird per pen and clotted in a serum collection tube (Grainer Bio-One, Kremsmuenster, Austria) for 2 h. Subsequently, serum was obtained by centrifugation at 1000× g for 15 min. The collected serum samples were stored at −80 °C for further analyses. After blood collection, the broilers were euthanized by cervical dislocation. After removing blood and digesta with ice-cold PBS, liver and mid-jejunal tissue samples were snap-frozen in liquid nitrogen and stored at −80 °C for further analyses. Five-centimeter segments of the mid-duodenum, mid-jejunum, and mid-ileum were immersed and stored in a 10% formaldehyde solution for fixation.

### 2.3. Serum Biochemistry

Serum biochemical markers were analyzed according to Castro and Kim [12]. The serum samples (100 μL) were added to Avian/Reptilian Profile Plus rotors (Abaxis, Union City, CA, USA), and the rotors were loaded into VetScan VS2 (Abaxis).

### 2.4. Glutathione Peroxidase (GPx) and Superoxide Dismutase (SOD) Activities

Around 100 mg of liver samples were homogenized in the designated solution according to the manufacture’s protocol using a bead beater (Biospec Products, Bartlesville, OK, USA) for 20 sec, and then centrifuged at 10,000× *g* for 15 min at 4 °C. Aliquot of supernatant was taken for the analyses of protein content using Pierce™ BCA Protein Assay Kits (Thermo Fisher Scientific, Rockford, IL, USA) after 10 times dilution. Samples were diluted 10 times and 400 times with sample buffer for GPx and SOD analyses, respectively. Afterwards, the analyses were conducted using Caymans GPx assay kit and Cayman SOD assay kit (Cayman Chemical, Ann Arbor, MI, USA), respectively, according to the manufacturer’s protocol. The activities of GPx were expressed as nmol/mg protein∙min, and SOD activities were shown as U/mg per protein.

### 2.5. Intestinal Morphology

The fixed tissues in 10% neutral-buffered formalin were embedded in paraffin and cut to 4 mm, and hematoxylin and eosin (H&E) staining were performed. The H&E-stained slides were pictured using a microscope (BZ-X810; Keyence, Osaka, Japan). The villus height (VH) and crypts depth (CD) of five well-oriented villi per section, and their corresponding crypts for the five villi were measured for duodenum, jejunum, and ileum samples, and CD was measured for ceca samples by using ImageJ (National Institutes of Health, Bethesda, MD, USA). The ratio of VH to CD was calculated for each villi and crypt.

### 2.6. Brush Border Digestive Enzyme Activities

The maximal enzyme activities (*Vmax*) of L-alanine aminopeptidase, maltase, and sucrase were determined. Approximately 100 mg of jejunum samples were homogenized in the PBS solution using the bead beater (Biospec Products) for 40 s and centrifuged at 3000× *g* for 15 min at 4 °C. The protein content of collected supernatant was determined using a Pierce BCA Protein Assay Kit (Thermo Fisher Scientific, Rockford, IL, USA). The activities of L-alanine aminopeptidase (EC. 3.4.11.2) and disaccharidases including sucrase (EC 3.2.1.48) and maltase (EC 3.2.1.20) were analyzed according to Maroux et al. [13] and Dahlqvist [14], respectively.

### 2.7. Statistical Analysis

Statistical analyses were performed using SAS (version 9.4; SAS Inst. Inc., Cary, NC, USA). To compare treatment means, one-way analysis of variance (ANOVA) using the general linear model (GLM), followed by Duncan’s test, was used. The significance was set at *p* < 0.05, and trends (0.05 ≤ *p* ≤ 0.1) were also presented.

## 3. Results

### 3.1. Growth Performance

The results of growth performance are shown in Table 2. In the starter phase, the PC, EO and OA + EO groups had a significantly lower FCR compared to the NC broilers. In the grower phase, the PC broilers tended to have higher BW compared to the NC, OA, and EO (*p* = 0.085). In the grower phase, broilers fed PC had the lowest FCR among treatments (*p* < 0.05). On D 42, the BW of PC and OA groups tended to be higher compared to the EO group (*p* = 0.055). The ADG of the OA broilers was significantly higher than the EO group in the finisher phase. In the finisher phase, the OA group tended to have a lower FCR compared to the PC, EO and OA + EO groups (*p* = 0.074). During the whole experimental period, the EO group tended to have a lower ADG compared to the PC and OA broilers (*p* = 0.054), whereas the OA group had a comparable ADG to the PC group and numerically higher ADG compared to the NC group.

### 3.2. Serum Biochemistry

As shown in Table 3, the levels of serum globulins (GLOB) of the PC and OA groups were significantly higher than the EO group (*p* < 0.05). The OA group tended to have higher GLOB:Albumin (ALB) compared to the EO group (*p* = 0.052).

### 3.3. Activities of Glutathione Peroxidase (GPx) and Superoxide Dismutase (SOD)

No significance differences were observed in the GPx and SOD activities among the treatments (*p* > 0.1; Table 4).

### 3.4. Intestinal Morphology

As shown in Table 5, the VH:CD ratio tended to be higher in the OA group compared to the EO group (*p* = 0.089). However, no differences were observed in the duodenum and ileum morphology (*p* > 0.1).

### 3.5. Activities of Brush Border Digestive Enzymes

There were no differences in the activities of L-alanine aminopeptidase, sucrase, and maltase in the jejunum among treatments (Table 6; *p* > 0.1).

## 4. Discussion

The purpose of study was to evaluate the effects of OAs and EOs on growth performance, serum biochemistry, antioxidant enzyme activities, intestinal morphology, and digestive enzyme activities as antibiotic alternatives in broiler chickens. In the starter phase, the improved feed efficiency of PC, EO, and OA + EO in the current study may indicate improved nutrient digestion and absorption in animals [15]. Potentially, improved nutrient digestion and absorption in the starter phase could be closely associated with the antibacterial effects of BMD and EOs [16,17]. The EOs (cinnamaldehyde, carvacrol, thymol and eugenol) used in the present study are known to have strong antimicrobial effects [18,19]. Young chickens are vulnerable to the bacterial infection, which potentially reduce the nutrient digestion and absorption capacity (e.g., *Escherichia coli* and *Salmonella* spp.) [20,21]. Hence, the antibacterial effects of EOs would increase the feed efficiency of broilers in the starter phase by reducing nutrient competition with pathogenic bacteria, decreasing inflammation, and enhancing short-chain fatty acid production [22]. A previous study by Pirgozliev et al. [23] also showed that supplementation of EOs, including carvacrol, cinnamaldehyde and capsicum oleoresin, improved feed efficiency in broiler chickens. However, in the current study, supplemental EOs did not improve growth performance compared to the NC group in the finisher phase. These data indicate that the dosage levels and supplementation period of the EOs should be adjusted in the finisher phase to maximize its effects for broilers. Depending on the dosage, plant products (EOs) can have beneficial or negative effects on animals [24], whereas supplemental OAs did not affect growth performance in the starter and grower phases. However, supplemental OAs enhanced feed efficiency compared to the PC group with similar a BW in the finisher phase in the current study. OAs could potentially improve nutrient utilization by modulating the pH of the gastrointestinal tract and exhibiting antimicrobial effects [25].

Serum biochemical indicators represent the general health status of broilers. The GLOB levels could be indicators for hepatic functions, nutrient utilization, and immune system, because GLOBs are produced in the liver and by the immune system. Moreover, GLOBs play an important role in antigen removal and nutrient transportation in animals [26]. A higher GLOB:ALB ratio represents the broilers’ augmented immune system [27]. Our study showed that the supplementation of BMD increased the GLOB levels compared to the lowest group (EOs), which is consistent with a previous study [27]. The OA group had the highest GLOB and GLOB:ALB values among the treatments, which suggests that OA supplementation improved the hepatic functions and immune system of broilers. Potentially, an improved hepatic function and immune system would be associated with the antimicrobial effects of OA in the gastrointestinal tract of chickens. However, the EO group had the numerical lowest GLOB and GLOB:ALB levels among the treatments, suggesting that supplemental EO at high dosages can compromise the liver function and immune system of the broilers in the finisher phase, while EO were generally known to improve liver function and immune system in chickens [28]. Nevertheless, these modulations in hepatic indicators did not lead to a change in the activities of GPx and SOD in the liver of broilers in the current study. These serum biochemical indicators corresponded to the growth performance of the broilers in this study, showing that these indicators are closely associated with broiler growth.

The OA group had the numerically highest ileal VH:CD among treatments in the current study. A higher VH:CD indicates improved nutrient digestion and absorption in the gut of the small intestine [29], because a longer VH indicates a larger intestinal surface for augmented nutrient digestion and absorption and a shorter CD means that the gut is more mature [30]. While jejunum is the main area for nutrient digestion and absorption, ileum still has an important role in absorbing end products [31]. Benzoic acid, formic acid, and lactic acid are known to improve intestinal morphology mainly by exhibiting antimicrobial effects in the gastrointestinal tract of chickens [30,32,33]. Results were observed in the ileum, the lower part of the gastrointestinal tract, potentially because most of the pathogens inhabit the lower gastrointestinal tract.

## 5. Conclusions

While supplemental 300 mg/kg EO and a combination of 2000 mg/kg OA and 300 mg/kg EO showed beneficial effects in feed efficiency in the starter phase, their beneficial effects on growth performance and gut health were not shown in the finisher phase. Supplementation of 2000 mg/kg OA improved feed efficiency, GLOB concentration, and the ileal morphology of broilers in the finisher phase as effectively as AGP. The supplementation of EO and OA could be an effective strategy to replace AGP in the starter and finisher phases, respectively.

## Figures and Tables

**Table 1:** Ingredients and nutrient compositions of basal diets (As-fed basis) ^1^.

Items	D 0 to 14	D 14 to 28	D 28 to 42
Ingredients (kg/ton)			
Corn	636.11	692.74	711.09
Soybean meal (480 g crude protein/kg)	320.37	259.93	235.96
Defluorinated phosphate	15.65	13.03	13.22
Filler ^1^ (sand or feed additives)	5.00	5.00	5.00
Soybean oil	4.95	11.79	17.16
Limestone	6.92	6.71	6.73
DL-Methionine 99%	2.88	2.63	2.46
L-Lysine HCl 78%	2.04	2.06	2.20
Vitamin Premix ^2^	2.50	2.50	2.50
Common Salt	1.47	1.83	1.83
L-threonine	0.80	0.48	0.54
Mineral Premix ^3^	0.80	0.80	0.80
Total	1000	1000	1000
Calculated energy and nutrient value, %			
Metabolizable energy, kcal/kg	3000	3100	3150
Crude protein	20.5	18	17
SID ^4^ Methionine	0.60	0.54	0.51
SID ^4^ Total sulfur amino acids	0.88	0.8	0.76
SID ^4^ Lysine	1.17	1.02	0.97
SID ^4^ Threonine	0.78	0.66	0.63
Total calcium	0.87	0.76	0.76
Available phosphate	0.44	0.38	0.38

^1^ Treatments including NC, negative control (no feed additives); PC, positive control (NC + 50 mg/kg bacitracin methylene disalicylate (BMD-50; Zoetis Products, Chicago Heights, IL, USA)); OA, organic acids [NC + 2000 mg/kg OA (Nutribins LLC, Walnut, CA, USA)], EO, essential oils (NC + 300 mg/kg EO (Nutribins LLC, Walnut, CA, USA)), and OA + EO (NC + 2000 mg/kg OA + 300 mg/kg EO) were formulated by adding sand and feed additives in the filler part (5000 mg/kg) of the basal diet as following: NC: 5000 mg/kg sand, PC: 4500 mg/kg sand + 500 mg/kg BMD-50, OA: 3000 mg/kg sand + 2000 mg/kg OA, EO: 4700 mg/kg sand + 300 mg/kg EO, and OA + EO: 2700 mg/kg sand + 2000 mg/kg OA + 300 mg/kg of EO. ^2^ Vitamin mix provided the following in mg/100 g diet: thiamine-HCl, 1.5; riboflavin 1.5; nicotinic acid amide 15; folic acid 7.5; pyridoxine-HCl, 1.2; d-biotin 3; vitamin B-12 (source concentration, 0.1%) 2; d-calcium pantothenate 4; menadione sodium bisulfite, 1.98; α-tocopherol acetate (source 500,000 IU/g), 22.8; cholecalciferol (source 5000,000 IU/g) 0.09; retinyl palmitate (source 500,000 IU/g), 2.8; ethoxyquin, 13.34; I-inositol, 2.5; dextrose, 762.2. ^3^ Mineral mix provided the following in g/100 g diet: Ca(H_2_PO_4_)_2_ · H_2_O, 3.62; CaCO_3_, 1.48; KH_2_PO_4_, 1.00; Na_2_SeO_4_, 0.0002; MnSO_4_ · H_2_O, 0.035; FeSO_4_ · 7H_2_O, 0.05; MgSO_4_ · 7H_2_O, 0.62; KIO_3_, 0.001; NaCl, 0.60; CuSO_4_ · 5H_2_O, 0.008; ZnCO_3_, 0.015; CoCl_2_ · 6H_2_O, 0.00032; NaMoO_4_ · 2H_2_O, 0.0011; KCl, 0.10; dextrose, 0.40. ^4^ SID: standard ileal digestible amino acid.

**Table 2:** Effects of individual or combination of organic acids and essential oils on growth performance parameters including body weight (BW), average daily gain (ADG, g/d), average daily feed intake (ADFI, g/d), feed conversion ratio (FCR, g/d), and mortality (%) of broiler chickens during the starter (D 0 to 14), grower (D 14 to 28), finisher (D 28 to 42), and whole period (D 0 to 42) phase.

Item	NC ^1^	PC	OA	EO	OA + EO	SEM	*p* Value
Initial BW (g)	44.33	44.31	44.41	44.43	44.41	0.24	0.883
**Starter**							
BW (g)	409.78	422.68	414.64	420.28	424.64	12.58	0.254
ADG	26.10	27.03	26.44	26.84	27.16	0.89	0.25
ADFI	36.74	36.5	36.52	36.55	37.00	1.02	0.9
FCR	1.41 ^a^	1.35 ^b^	1.38 ^ab^	1.36 ^b^	1.36 ^b^	0.02	<0.01
**Grower**							
BW (kg)	1.46 ^b^	1.54 ^a^	1.45 ^b^	1.45 ^b^	1.47 ^ab^	0.06	0.085
ADG	75.20 ^ab^	79.65 ^a^	74.21 ^b^	73.23 ^b^	75.05 ^ab^	3.76	0.061
ADFI	119.77	121.47	117.59	116.4	118	4.41	0.345
FCR	1.59 ^a^	1.53 ^b^	1.59 ^a^	1.59 ^a^	1.58 ^a^	0.04	0.035
**Finisher**							
BW (kg)	3.05 ^ab^	3.10 ^a^	3.10 ^a^	2.97 ^b^	2.98 ^ab^	0.09	0.055
ADG	113.55 ^ab^	111.7 ^ab^	117.78 ^a^	108.91 ^b^	107.55 ^b^	5.55	0.031
ADFI	186.01	188.47	184.77	186.77	182.21	6.07	0.45
FCR	1.64 ^ab^	1.69 ^a^	1.59 ^b^	1.69 ^a^	1.69 ^a^	0.07	0.074
**Whole period** **(D 0 to 42)**							
ADG	71.62 ^ab^	72.79 ^a^	72.81 ^a^	69.66 ^b^	69.92 ^ab^	2.26	0.054
ADFI	114.18	115.48	113.63	112.37	112.71	2.98	0.404
FCR	1.59	1.58	1.56	1.61	1.61	0.03	0.196

^1^ NC, negative control; PC, positive control (NC + 50 mg/kg bacitracin methylene disalicylate (BMD-50; Zoetis Products, Chicago Heights, IL, USA)); OA, organic acids (NC + 2000 mg/kg OA (Nutribins LLC, Walnut, CA, USA)); EO, essential oils (NC + 300 mg/kg EO (Nutribins LLC, Walnut, CA, USA)), and OA + EO (NC + 2000 mg/kg OA + 300 mg/kg EO). ^a,b^ Means within a row with no common superscripts represent significant differences (*p* < 0.05) analyzed using one-way ANOVA, followed by Duncan’s multiple range test.

**Table 3:** Effects of individual or combination of organic acids and essential oils on the serum biochemistry of broiler chickens on D 35.

Item	NC ^1^	PC	OA	EO	OA + EO	SEM	*p* Value
AST (U/L) ^2^	384.50	388.17	413.17	335.33	431.33	102.87	0.56
UA (mg/dL)	4.07	4.5	4.13	4.25	4.58	0.81	0.756
K^+^ (mM)	8.5	9.6	10.22	7.13	8.5	1.97	0.102
Na^+^ (mM)	153.33	154	151.83	148.17	155.17	7.68	0.571
Ca^+^ (mM)	11.8	12.13	11.71	11.30	11.88	0.88	0.588
Phos (mM)	5.98	6.12	5.85	5.73	5.47	0.64	0.481
GLU (mM)	243	257.83	248.67	248.5	254.83	26.7	0.875
TP (g/dL)	3.07	3.25	3.13	2.87	2.98	0.26	0.156
GLOB (g/dL)	0.65 ^ab^	0.75 ^a^	0.78 ^a^	0.5 ^b^	0.58 ^ab^	0.16	0.049
ALB (g/dL)	2.4	2.52	2.35	2.35	2.42	0.17	0.431
GLOB:ALB	0.27 ^ab^	0.3 ^ab^	0.33 ^a^	0.22 ^b^	0.24 ^ab^	0.07	0.052

^1^ NC, negative control; PC, positive control (NC + 50 mg/kg bacitracin methylene disalicylate (BMD-50; Zoetis Products, Chicago Heights, IL, USA)); OA, organic acids (NC + 2000 mg/kg OA (Nutribins LLC, Walnut, CA, USA)); EO, essential oils (NC + 300 mg/kg EO (Nutribins LLC, Walnut, CA, USA)), and OA + EO (NC + 2000 mg/kg OA + 300 mg/kg EO). ^2^ AST: aspartate aminotransferase, UA: uric acid, GLU: glucose, PHOS: Phosphorus, TP: total proteins, ALB: albumin, GLOB: globulins. ^a,b^ Means within a row with no common superscripts represent significant differences (*p* < 0.05) analyzed using one-way ANOVA followed by Duncan’s multiple range test.

**Table 4:** Effects of individual or a combination of organic acids and essential oils on the activities of glutathione peroxidase (GPx; nmol/mg protein∙min) and superoxide dismutase (SOD; U/mg per protein) in broiler chickens on D 35.

Item	NC ^1^	PC	OA	EO	OA + EO	SEM	*p* Value
GPx	124.96	125.16	115.67	135.31	126.47	15.39	0.324
SOD	8.59	7.64	9.20	8.26	8.78	3.35	0.945

^1^ NC, negative control; PC, positive control (NC + 50 mg/kg bacitracin methylene disalicylate (BMD-50; Zoetis Products, Chicago Heights, IL, USA)); OA, organic acids (NC + 2000 mg/kg OA (Nutribins LLC, Walnut, CA, USA)); EO, essential oils (NC + 300 mg/kg EO (Nutribins LLC, Walnut, CA, USA)), and OA + EO (NC + 2000 mg/kg OA + 300 mg/kg EO).

**Table 5:** Effects of individual or combination of organic acids and essential oils on the villus height (VH, μm), crypts depth (CD, μm), and VH:CD in the duodenum, jejunum, and ileum of broiler chickens on D 35.

Item	NC ^1^	PC	OA	EO	OA + EO	SEM	*p* Value
**Duodenum**							
VH	2534.9	2827.4	2910.5	2716.3	2626.1	345.87	0.361
CD	267.25	290.69	294.77	310.05	277.94	46.22	0.566
VH:CD	2.58	2.58	2.61	2.6	2.53	0.12	0.812
**Jejunum**							
VH	1710.3	1697.2	1676.7	1652.7	1763.4	205.62	0.916
CD	250.11	260.23	241.23	267.36	269.66	42.23	0.759
VH:CD	2.51	2.58	2.65	2.56	2.60	0.1	0.274
**Ileum**							
VH	1129.8	1097.03	1152.9	1114.76	1055.81	115.97	0.668
CD	218.33	222.83	229.03	230.1	193.43	26.35	0.137
VH:CD	2.53 ^ab^	2.48 ^ab^	2.65 ^a^	2.46 ^b^	2.47 ^ab^	0.12	0.089

^1^ NC, negative control; PC, positive control [NC + 50 mg/kg bacitracin methylene disalicylate (BMD-50; Zoetis Products, Chicago Heights, IL, USA)]; OA, organic acids [NC + 2000 mg/kg OA (Nutribins LLC, Walnut, CA, USA)]; EO, essential oils [NC + 300 mg/kg EO (Nutribins LLC, Walnut, CA, USA)], and OA + EO (NC + 2000 mg/kg OA + 300 mg/kg EO). ^a,b^ Means within a row with no common superscripts represent significant differences (*p* < 0.05) analyzed using one-way ANOVA followed by Duncan’s multiple range test.

**Table 6:** Effects of individual or combination of organic acids and essential oils on the activities of L-alanine aminopeptidase (nmol p-nitroaniline liberated/mg protein∙min), sucrase (nmol glucose released/mg protein∙min), and maltase (nmol glucose released/mg protein∙min) in the jejunum of broiler chickens on D 35.

Item	NC ^1^	PC	OA	EO	OA + EO	SEM	*p* Value
L-alanine aminopeptidase	10.17	11.11	7.81	11.38	10.91	5.6	0.942
Sucrase	1.72	1.51	1.45	1.33	1.62	0.46	0.624
Maltase	17.16	15.41	15.09	13.52	16.96	3.6	0.419

^1^ NC, negative control; PC, positive control (NC + 50 mg/kg bacitracin methylene disalicylate (BMD-50; Zoetis Products, Chicago Heights, IL, USA)); OA, organic acids (NC + 2000 mg/kg OA (Nutribins LLC, Walnut, CA, USA)); EO, essential oils (NC + 300 mg/kg EO (Nutribins LLC, Walnut, CA, USA)), and OA + EO (NC + 2000 mg/kg OA + 300 mg/kg EO).

## Data Availability

Not applicable.
